# Dispensing of high concentration Ag nano-particles ink for ultra-low resistivity paper-based writing electronics

**DOI:** 10.1038/srep21398

**Published:** 2016-02-17

**Authors:** Fuliang Wang, Peng Mao, Hu He

**Affiliations:** 1 State Key Laboratory of High Performance Complex Manufacturing, Changsha 410083, China; 2 School of Mechanical and Electrical Engineering, Central South University, Changsha 410083, China

## Abstract

Paper-based writing electronics has received a lot of interest recently due to its potential applications in flexible electronics. To obtain ultra-low resistivity paper-based writing electronics, we developed a kind of ink with high concentration of Ag Nano-particles (up to 80 wt%), as well as a related dispensing writing system consisting an air compressor machine and a dispenser. Additionally, we also demonstrated the writability and practical application of our proposed ink and writing system. Based on the study on the effect of sintering time and pressure, we found the optimal sintering time and pressure to obtain high quality Ag NPs wires. The electrical conductivity of nano-silver paper-based electronics has been tested using the calculated resistivity. After hot-pressure sintering at 120 °C, 25 MPa pressure for 20 minutes, the resistivity of silver NPs conductive tracks was 3.92 × 10^−8^ (Ωm), only 2.45 times of bulk silver. The mechanical flexibility of nano-silver paper-based electronics also has been tested. After 1000 bending cycles, the resistivity slightly increased from the initial 4.01 × 10^−8^ to 5.08 × 10^−8^ (Ωm). With this proposed ink preparation and writing system, a kind of paper-based writing electronics with ultra-low resistivity and good mechanical flexibility was achieved.

With the rapid development of microelectronics, the corresponding electronic components also showed a lightweight, three-dimensional, flexible trend^1^,^2^,^3^. To meet the requirement of folded and unfolded print circuit, a lot of work has been carried out on flexible substrates for flexible electronics, and generated many new applications including flexible displays^4^,^5^, antenna arrays^6^, electronic solar cell arrays^7^, radio-frequency identification (RFID) tags^8^,^9^, batteries^10^,^11^, and biomedical devices^12^. As a substrate for fabricating flexible electronics, paper-based electronics not only is lightweight, biodegradable, and recyclable, but also can be rolled or folded, which shows a great potential on the application of flexible electronics^6^,^13^,^14^.

Due to the compatibility with high conductivity and low-temperature processing required by paper substrate, nano-particle (NP) metal conductive inks present a promising candidate for large-area paper-based flexible electronic applications, and have been commonly applied in functional printing based on the high electrical properties^15^,^16^,^17^. Among the metal NPs, Ag NPs ink has been employed most extensively because of its excellent electrical conductivity and oxidation stability^18^,^19^,^20^.

Regarding the preparation and printing method of the nano-silver conductive ink, lots of research has been done. To date, functional printing approaches such as inkjet printing^20^,^21^, airbrush spraying^22^ or sputter coating^23^, were used to deposit nano-silver conductive ink. However, these approaches were limited by many factors, such as materials waste, complex manufacturing process, expensive equipment, high costs, and environmental pollution, etc. To overcome those disadvantages, Lewis *et al.*^6^ demonstrated a facile pen-on-paper approach that offers a low-cost, portable fabrication method for printed electronic and optoelectronic devices by directly writing patterns on paper with a rollerball pen. Since then, many other researches have been carried out based on this approach. In 2012, Yang *et al.* deployed a new uniform organic silver ink with 20 wt% solid loading of Ag NPs to write directly on a sulfuric paper substrate and formed conductive connection at 200 °C for 60 minutes, which obtained the resistivity as 3.26 × 10^−6^ (Ωm)^24^. In 2013, Liu *et al.* studied Ag NPs (20 wt%) ink writing on sulfuric paper and the silver nano-ink was sintered at a temperature of 180~220 °C with resistivity of 2.1 × 10^−6^ (Ωm)^25^. In 2013, Xu *et al.* directly wrote Ag NPs (20 wt%) ink on Epson photo paper and sintered at optimum hot-pressure condition (temperature of 120 °C/pressure of 25 MPa/time of 15 minutes), and obtained a relative low resistivity value of 1.43 × 10^−7^ (Ωm)^26^. However, to prevent the occurrence of clogging, rollerball pen writing implementation always limited the solid loading of Ag NPs in hybrid ink low to 20~45 wt%, and lots of organic solvents, like ethanol, glycerol, and ethylene glycol were required to improve the viscosity. Theoretically, the low solid loading of Ag NPs would decrease the thickness of silver tracks, leading to high resistance, and large amounts of organic solvents also increase the contact resistance between the nanoparticles. Thus consequently, the electrical and mechanical properties of the designed sample could degrade significantly.

As a writing method for ink or glue, time-pressure dispensing technology has been widely employed in such processes as advanced integrated circuit encapsulation (AICE) and surface mount technology (SMT) in the semiconductor industry^27^,^28^,^29^, due to its flexibility to ink/glue viscosity, simple operation and easy maintenance, which make it the great potential for writing application with high concentration of Ag NPs ink.

In order to obtain an ultra-low resistivity writing electronics, a kind of high concentration Ag nano-particles conductive ink containing Ag NPs up to 80 wt% was prepared in the paper. Additionally, a time-pressure dispenser was employed as the writing device to overcome the clogging problem caused by high concentration Ag NPs ink, and patterns were written on Epson photo paper (Type: C13S042551, cast coated photo paper). Given that the rough surface of common printing paper could prevent the continuity of the conductive films and thus lead to a high resistivity, therefore, cast coated photo paper that has a coating of silicon dioxide NPs and forms tiny inorganic–organic hybrid fine particles which can enhance the adhesion strength of the conductive film is employed in this work. After that, hot-pressure sintering was used to convert the writing patterns into its conductive counterparts. Finally, the conductivity, mechanical flexibility and adhesion of the writing electronics were validated.

## Results

### Effect of sintering time on resistivity

To study the effects of sintering time on the resistivity of tracks with high solid loading of Ag NPs ink, a series of silver tracks was dispensed on Epson photo paper with the width of about 1 mm, thickness of about 4 μm and a length of about 45 mm. The tracks was dried 2 hours at room temperature in air, then the samples were sintered using pressure of 25 MPa at 120 °C for different sintering time with 2, 5, 8, 11, 14, 17 and 20 minutes, respectively.

**Figure 1:**
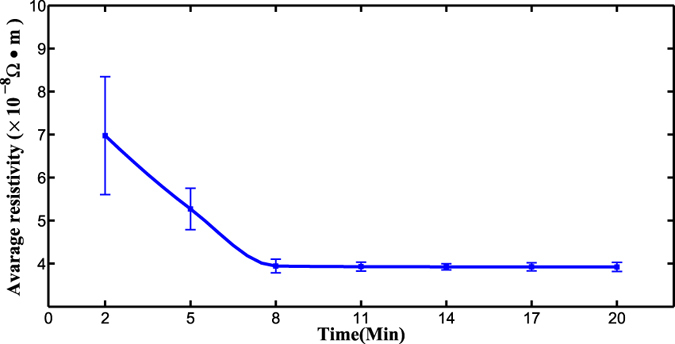
Average resistivity of sliver tracks with different sintering time with temperature as 120 °C and pressure as 25 MPa.

Figure 1 shows the average resistivity of silver tracks that sintered with different time. It shows that the resistivity decreased with the increasing of sintering time. At the beginning (from 2 to 8 minutes), the resistivity rapidly decreased from 6.97 × 10^−8^ to 3.94 × 10^−8^ (Ωm). After 8 minutes, the resistivity approximately approached a stable value regardless of the sintering time, which only decreased from 3.94 × 10^−8^ to 3.92 × 10^−8^ (Ωm) from 8 to 20 minutes. Therefore, 8 minutes seems to be the optimal hot-pressure sintering time in this study. Compared with the optimal sintering time as 15 minutes in Xu *et al.*^26^ under the same sintering pressure and temperature, we achieved a faster sintering process with 7 minutes less. Figure 1 also shows that the lowest resistivity is 3.92 × 10^−8^ (Ωm), which is only 2.45 times of bulk silver (1.58 × 10^−8^ Ωm). Furthermore, unlike previous researches that used rollerball pen and relatively low concentration nano-silver conductive ink^3^,^17^,^20^, the preparation and writing method of nano-silver ink described in this paper can greatly improve electrical properties of nano-silver tracks. The ultra-low resistivity and short sintering time were subject to the less solvent and higher solid loading of Ag NPs in ink in our work.

### Effect of pressure on resistivity

Pressure was demonstrated on accelerating the sintering process of nano-silver tracks, lowering the sintering temperature, and decreasing the sintering time. To study the effects of pressure on the resistivity of tracks with high solid loading of Ag NPs ink, a group of nano-silver tracks were produced and sintered with different pressure at 120 °C for 8 minutes. To prevent permanently deformation of paper during sintering, the applied pressure was set between 0 and 30 MPa.

**Figure 2:**
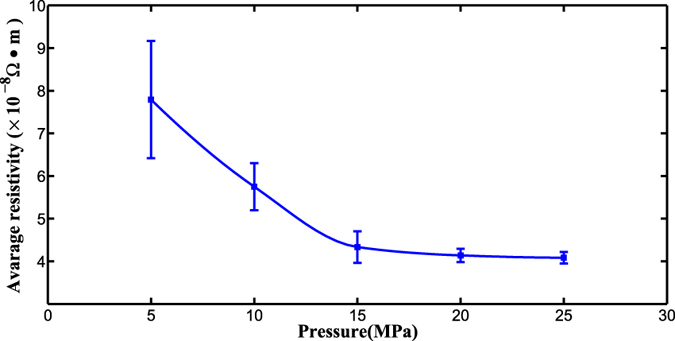
Average resistivity of sliver tracks with different sintering pressure with temperature as 120 °C and time as 8 *min*.

Twenty-five samples were produced in each experiment condition, and the resistivity of all samples were measured. The results are shown in Fig. 2, which shows that the average resistivity of sample follows a downward trend with the growth of the sintering pressure. When a pressure of 5 MPa was applied, the resistivity of sample was 7.79 × 10^−8^ (Ω. m), only 3.8 times of bulk silver. While the pressure increased to 20 MPa, the resistivity of sample decreased to 4.13 × 10^−8^ (Ω. m). However, the resistivity keeps the stable even with the growth of pressure. Thus, an optimal pressure in this study is 20 MPa.

**Figure 3:**
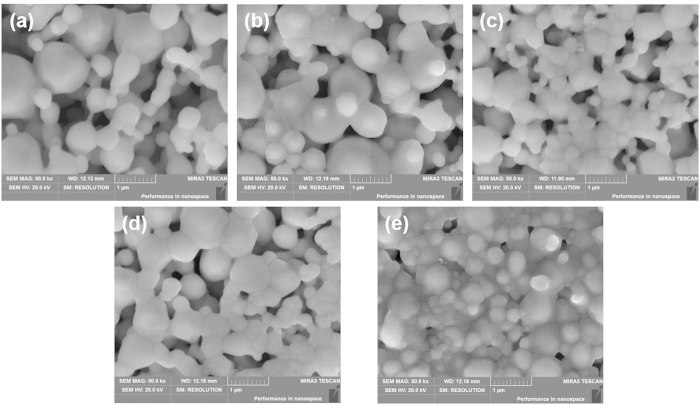
SEM images of nano-silver tracks sintered at temperature 120 °C for 8 minutes with different pressure. (**a**) 5 Mpa (**b**) 10 Mpa (**c**) 15 Mpa (**d**) 20 Mpa (**e**) 25 Mpa.

To verify the effect of pressure on the electrical properties of the samples, we observed the different morphologies of the written tracks with different sintering pressure using SEM. The results obtained are shown in Fig. 3(a–e), it can be found that the porosity decreased with the increase of sintering pressure. With the pressure of 5 MPa, only a few Ag NPs were merged together to form a large ball with the size about 200~400 nm, and those large balls only have limited bridge connections. In other word, voids between those balls occupied quite large volume ratio. Therefore relative higher resistivity was obtained in this condition. With the applied pressure increased, the bridge connection between large balls gradually increased and void gradually decreased accordingly. When the pressure increased to 20 MPa, almost all Ag NPs were merged together, and only a few of voids can be observed. When the pressure comes to 25 MPa, the Ag NPs distributed uniformly and void disappeared completely.

### Mechanical properties of nano-silver conductive tracks

**Figure 4:**
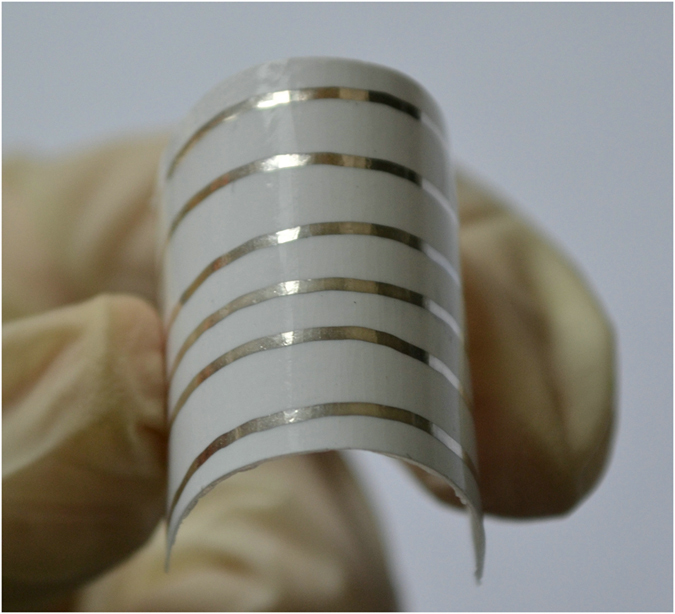
Photograph of a written pattern on paper substrate bending outwards by nearly 90° at the hot-pressure sintering condition with temperature as 120 °C, pressure as 25 MPa, and sintering time as 15 *min.*

To demonstrate the mechanical flexibility of the silver tracks produced by dispenser and hot-pressure sintering, a set of tracks on paper substrate was produced and sintered with a temperature of 120 °C and pressure of 25 MPa for 15 minutes. Then, the resistivity of tracks before and after bending cycles was measured, respectively. We manually held the two ends of the strap with Ag NPs tracks and repeated the bending movements. The bending angle was kept around 90° using a right-angle ruler as the reference, as shown in Fig. 4. The resistivity of sample was recorded with the bending cycle number of 0, 100, 200, 300, 400, 500, 600, 700, 800, 900 and 1000.

**Figure 5:**
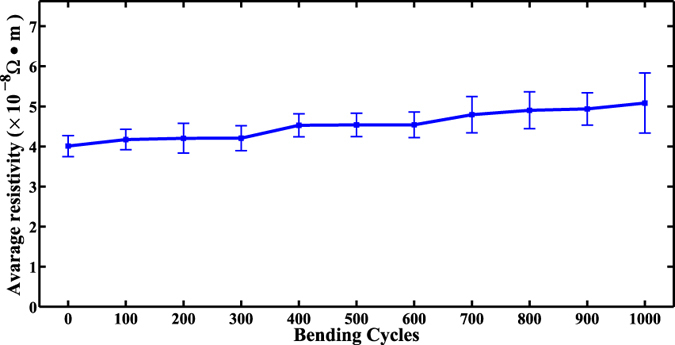
The average electrical resistivity of sample after bending cycles (120 °C, 25 Mpa, 15 *min*).

Five experiments were carried out in each experimental condition, and the experimental results are shown in Fig. 5. It illustrates that the average resistivity of sample increased slightly with the bending cycles, but always stayed at a relative low level. After 1000 bending cycles, the resistivity only increased from the initial 4.01 × 10^−8^ to 5.08 × 10^−8^ (Ω .m), which indicates that the sample has a good performance on mechanical flexibility.

**Figure 6:**
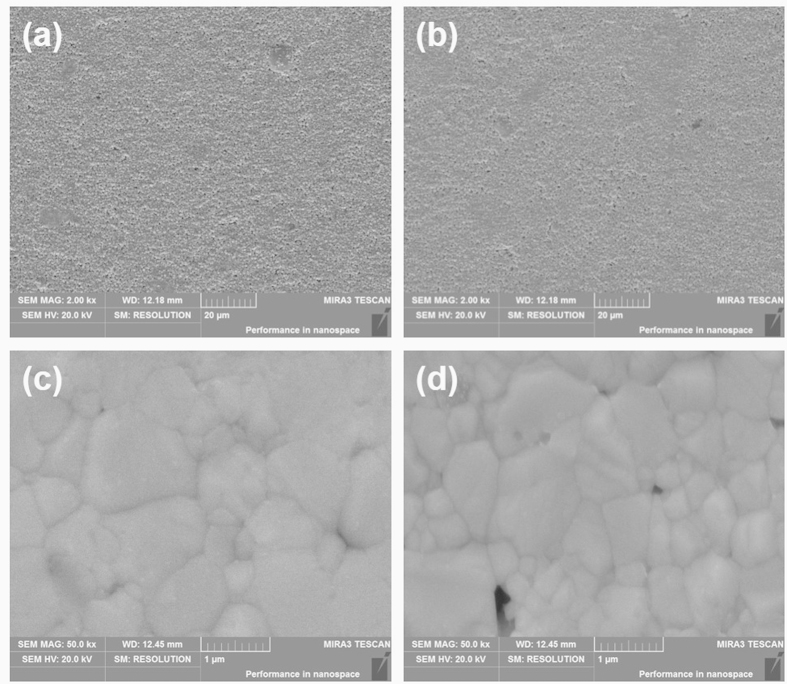
SEM images of silver tracks by hot-pressure sintering (120 °C, 25 Mpa, 15 *min*). (**a**,**b**) illustrate the SEM microstructure image of Ag tracks before and after bending cycles, respectively. (**c**,**d**) are the magnified images of the silver tracks before and after bending cycles.

To further verify the mechanism of mechanical flexibility of the designed samples, we observed the different morphologies of the written tracks before and after bending using SEM, as shown in Fig. 6. It can be observed that the tracks after bending were still uniform and compact as before, which also indicated that the electronics produced by the proposed method in this study has good mechanical flexibility. Additionally, Fig. 6(c,d) illustrate few slight cracks in the microstructure of Ag tracks after bending cycles which can explain the slight increase of electrical resistivity of Ag tracks.

### Application

**Figure 7:**
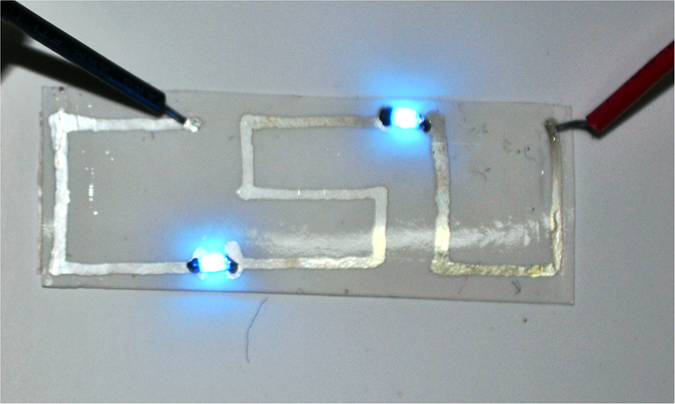
Photograph of an electronic circuit drawn by dispenser with solid loading of Ag NPs ink to 80 wt%, and sintered at 120 °C and 20 MPa for 8 *min.*

Finally, a simple electronic circuit on paper, as shown in Fig. 7, was made to demonstrate the application for writing electronics. The circuit contained three segments of silver tracks like the letters of ‘C’, ‘S’ and ‘U’ which are short for authors’ affiliation Central South University, and two surface-mounted LED were connected in series by these tracks. The conductive tracks were drawn by dispensing 80 wt% solid loading of Ag NPs ink on Epson photo paper, and tracks were sintered at 120 °C with 20 MPa for 8 minutes. LEDs were placed in tracks gaps and welded to the tracks using solder paste. After the circuit powered by a direct-current power supplier (Agilent B2901A), the LEDs are illuminated and works well, which indicated that Ag NP ink with solid loading of up to 80 wt% can be directly written by dispenser to fabricate paper-based electronics with low resistivity and high flexibility.

## Discussion

With the aim of obtaining low resistivity paper-based writing electronics, a high solid loading of Ag NPs ink (up to 80 wt%) has been prepared, and a novel dispensing writing system consisting an air compressor machine and a dispenser has been developed. Additionally, the writability and practical application have been validated. The electrical conductivity and mechanical flexibility of paper-based circuit have been tested. After hot-pressure sintering at 120 °C, 25 MPa pressure for 20 minutes, the resistivity of silver conductive tracks was 3.92 × 10^−8^ (Ωm), only 2.45 times of bulk silver. A rigorous ink preparation and designed writing method can not only significantly reduce the sintering time, but also can improve electrical and mechanical performance even under low sintering pressure. Furthermore, the additives in the Ag NPs ink are volatilized after sintering process based on basic EDX analysis, which in turn contributes to the decrease of electric resistivity of Ag tracks. Bending test shows that electronics using our prepared Ag NPs still has robust mechanical flexibility after 1000 bending cycles. Based on these excellent properties, a simple LED lighting electronic circuit was fabricated, which demonstrated that the ink preparation and writing method proposed in this paper are promising for writing electronics.

## Methods

### Material

Nano-silver particles with purity of 99.9% and average size of 20 nm were purchased from China Shanghai Chao Wei Technology Co., Ltd. The organic solvents like glycerol, ethylene glycol and absolute ethyl alcohol were purchased from China Tianjin Yongda Chemical Co. Ltd. All of these were used without additional purification.

### Preparation of nano-silver conductive ink

The preparation process goes on as follows: Firstly, the nano-silver particles were dispersed in the mixture solvent of ethanol, ethylene glycol and glycerol using the ultrasonic bathing for 10 minutes, the volume ratio of solvent was ethanol: ethylene glycol: glycerol = 60:35:5%. Then, the mixture solution was held for 10 minutes to settle down the Ag NPs, and the top transparent solution in the tube was removed to increase the solid loading of Ag NPs. Finally, 5 wt% glycerol was added into the solution to improve the wettability and surface tension of ink, and ultrasonic agitation was applied again until the ink becomes uniform. The obtained conductive ink can reach the solid loading of Ag NPs as high as 80 wt%.

### Writing system setup

**Figure 8:**
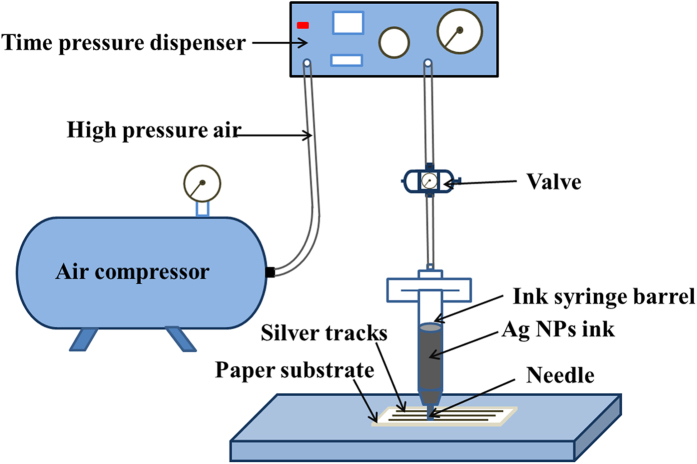
Schematic of the writing system.

In order to write patterns on a paper substrate using the high concentrative Ag NPs ink, a writing system consisting an air compressor machine and a dispenser was developed, as shown in Fig. 8. Firstly, the ink syringe barrel and needle were cleaned thoroughly in ethanol using ultrasonic bath, and dried with clean air. Then, the Ag NPs ink was filled into the ink syringe barrel, and the ink syringe barrel was connected to the dispenser with needle installed. Finally, the air pressure of dispenser was adjusted together with the valve open to dispense the Ag NPs ink on the Epson photo paper to form desired tracks. Note that the dispenser tip was controlled automatically using servo motor via LabView tool to generate uniformal silver tracks.

### Hot pressing sintering

**Figure 9:**
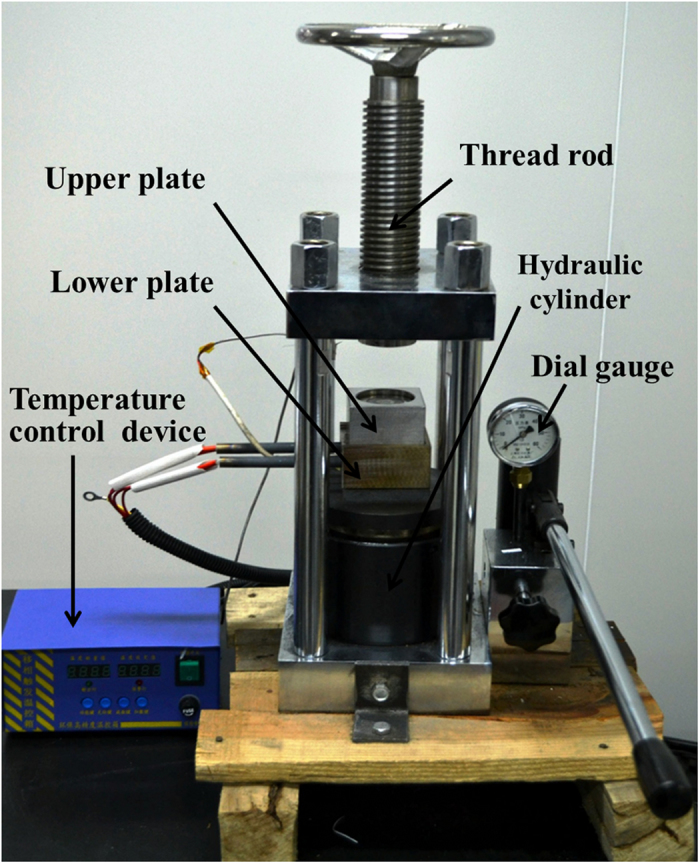
Hot pressing sintering system.

To convert the non-conductive writing pattern into its conductive counterparts, the hot pressing sintering method was used. The hot pressing device is composed by a temperature control device and a pressure loading device, as shown in Fig. 9. The temperature control device can heat the lower plate to 350 °C with the speed of 300 °C/*min* and accuracy of ± 0.5 °C. The pressure device can provide a maximum pressure up to 30 MPa.

**Figure 10:**
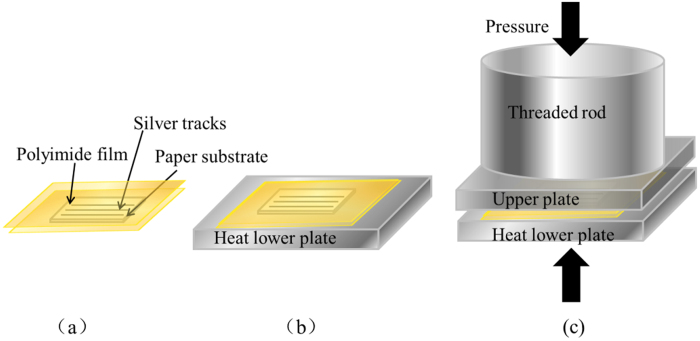
Schematic of hot pressing sintering.

The hot pressing sintering process is illustrated in Fig. 10. Firstly, the paper with silver tracks was covered by as-received polyimide films (0.05mm thickness) synthesized using pyromellitic dianhydride (PMDA) and diaminodiphenyl ether (DDE), which is resistant to thermal deformation and is easy to separate with Ag NPs tracks even under high temperature on both sides to prevent the sample from being contaminated by the heating plate. Then, the sample was placed on the lower plate which was preheated to the sintering temperature, meanwhile covered by the upper plate. In addition, the thread rod was manually adjusted until it reached the upper plate. Finally, pressure was applied using the hydraulic cylinder system and controlled via dial gauge manually.

### Instruments

After sintering, the surface morphology of nano-silver tracks on paper was observed by Scanning Electron Microscopy (SEM, TES Scan MIRAS LMQ). The resistance of tracks was measured by Intelligent DC Low Resistance Meter (Changzhou City, Jinai Electronic Technology Co., Ltd.) using four-probe method, which can measure the resistance between 1 μΩ and 20 kΩ, with the accuracy of ± 0.2%. The electronic circuit was powered by Agilent B 2901A (Agilent Technologies Singapore Pte. Ltd.).

### Calculation of electrical resistivity

The resistivity of tracks was calculated in accordance with its experimentally measured resistance. The electrical resistivity was calculated as


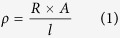


where R is the resistance, *l* is length and *A* is the cross-sectional area of the track, it was given as


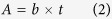


where *b* is the width and *t* is thickness of Ag NPs track. Therefore, the resistivity (ρ) of the track can be calculated as


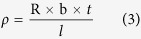


Given that R can be measured by the resistance meter, b as well as l can be measured by Vernier caliper, and t can be measured by SEM, therefore the resistivity can be obtained by equation (3). According to the experiment, typical *R* = 0.45 Ω, *b* = 1 mm, *t* = 4 μm, *l* = 45 mm, thus a typical resistivity value measured in this study is 4.13 × 10^−8^ (Ωm), which is only 2.60 times greater than the value of bulk silver.

## Additional Information

**How to cite this article**: Wang, F. *et al.* Dispensing of high concentration Ag nano-particles ink for ultra-low resistivity paper-based writing electronics. *Sci. Rep.* **6**, 21398; doi: 10.1038/srep21398 (2016).
